# TomoPy: a framework for the analysis of synchrotron tomographic data

**DOI:** 10.1107/S1600577514013939

**Published:** 2014-08-01

**Authors:** Doǧa Gürsoy, Francesco De Carlo, Xianghui Xiao, Chris Jacobsen

**Affiliations:** a Advanced Photon Source, Argonne National Laboratory, 9700 South Cass Avenue, Argonne, IL 60439-4837, USA

**Keywords:** tomography, X-ray imaging, phase retrieval

## Abstract

Analysis of tomographic datasets at synchrotron light sources (including X-ray transmission tomography, X-ray fluorescence microscopy and X-ray diffraction tomography) is becoming progressively more challenging due to the increasing data acquisition rates that new technologies in X-ray sources and detectors enable. The next generation of synchrotron facilities that are currently under design or construction throughout the world will provide diffraction-limited X-ray sources and are expected to boost the current data rates by several orders of magnitude, stressing the need for the development and integration of efficient analysis tools. Here an attempt to provide a collaborative framework for the analysis of synchrotron tomographic data that has the potential to unify the effort of different facilities and beamlines performing similar tasks is described in detail. The proposed Python-based framework is open-source, platform- and data-format-independent, has multiprocessing capability and supports procedural programming that many researchers prefer. This collaborative platform could affect all major synchrotron facilities where new effort is now dedicated to developing new tools that can be deployed at the facility for real-time processing, as well as distributed to users for off-site data processing.

## Abstract 2

A collaborative framework for the analysis of synchrotron tomographic data which has the potential to unify the effort of different facilities and beamlines performing similar tasks is described. The proposed Python-based framework is open-source, platform- and data-format-independent, has multiprocessing capability and supports functional programming that many researchers prefer.

## 1. Introduction   

Analysis of tomographic datasets at synchrotron light sources including X-ray transmission tomography (XTT), X-ray fluorescence microscopy (XFM) and X-ray diffraction tomography (XDT) (Banhart, 2008 ▶) is becoming progressively more challenging due to the increasing data acquisition rates that new technologies in X-ray sources and detectors enable. The next generation of synchrotron facilities that are currently under design or construction throughout the world will provide diffraction-limited X-ray sources and are expected to boost the current data rates by several orders of magnitude, thereby stressing the need for the development and integration of automated and efficient analysis tools more than ever.

It is often the case that researchers collect data at different facilities to take advantage of specific instrument characteristics and specifications; however, they can then face difficulties in integrating this data using the various tools that different facilities provide. Because there are strong similarities amongst the instruments at various facilities, we have developed a software framework in which each facility and user group can utilize and contribute to, ultimately saving on-site resources by sharing the computing tasks with the user community.

Here we describe in detail our framework. The basic principles of this Python-based open-source framework, called TomoPy (http://www.aps.anl.gov/tomopy/), include ease of collaborative development of scripts, platform and data format independence, modularity, and support for a functional programming style that many researchers prefer. Python is used as an interface for an easy integration of codes written in different languages. In the following sections we will provide a brief background on the analysis of tomographic data at synchrotrons, and then shortly introduce the framework by describing the model structure together with the currently available methods.

## 2. Background   

When it comes to the digital storage of tomographic experimental data and the development of analysis tools at synchrotron light sources around the world, the situation is very heterogeneous. As different research teams and instruments have grown at various facilities, they have often developed local data and analysis models based on the instrument hardware specificity and often drawing upon the particular preferences of a scientist writing software.

Many tomographic analysis tools utilize licensed (and often expensive) software packages like Matlab (The MathWorks Inc.) and IDL (Exelis VIS), while others rely on specific (and often complex to maintain) computing infrastructure like MPI-based CPU or GPU clusters. This diversity and specialization hampers widespread delivery of an integrated development environment and limits its long-term use. On the other hand, modern chip design shows a clear trend toward integration of multiple processor cores, thus permitting concurrency without a need for a dedicated cluster. The availability of inexpensive multi-core CPU workstations and the reduced cost of computer memory allow one to considerably simplify the computing infrastructure required to process tomographic data (Rivers, 2012 ▶), so that single workstation tools are able to perform most computing tasks in reasonable times. Tools of this type also allow the analysis to be performed on an off-site location, freeing on-site computational resources for other tasks.

This transition is affecting all major synchrotron facilities where new effort is now dedicated into developing tools that can be deployed at the facility for real-time processing as well as distributed to users for off-site data processing. We believe this will pave the way for an efficient use of the existing knowledge and methods base, thereby fostering collaborations and development of novel methods.

## 3. TomoPy: a Python-based framework   

Choosing a coding language is critical to the integration of analysis methods but it is usually difficult to balance the cost and development efforts. For example, Matlab and IDL use coding practices which are based on matrix and linear algebra operations that many researchers find useful, but different laboratories choose to purchase one package over another so that inevitably some researchers are left out when a choice to support one of these packages is made. The statistical programming language *R* (http://www.r-project.org) is an open-source alternative, but it is generally considered as having a steep learning curve and is usually difficult to maintain for large projects. Low-level programming languages like C or Fortran provide the desired computational efficiency but lack in readability and may hinder a collaborative code development. Python plus standard packages like NumPy and SciPy offer a free, open-source, modular, readable and manageable framework that researchers can use and contribute to easily. Python also offers easy integration to C or Fortran codes through shared libraries in situations where computation speed is critical. In addition, the native control software running at several synchrotron facilities, EPICS (http://www.aps.anl.gov/epics/), is accessible *via* Python, allowing simultaneous data analysis and real-time feedback on the instrumentation status. These features make Python the tool of scripting language for development framework.

Digestion of synchrotron tomographic data is essentially modular in nature such that the processes can be divided into a number of independent, and in many situations serialized, steps. A natural way of modularizing these processes is to group tasks according to the similarities in transformations applied on data, that is, distributing tasks into modules like pre-processing (sinogram-to-sinogram transformations), reconstruction (sinogram-to-image transformations) and post-processing (image-to-image transformations). Each module can have a number of different sub-modules specific to targeted application (*e.g.* XTT, XFM, XDT). This modular tree design of data pipeline enables a degree of control for the entire process by checking the quality of individual steps, allows inheritance of common methods available to different tomographic techniques, and at the same time improves the readability of the code. Besides, the data-driven pipeline of tomographic reconstruction allows for an easy kind of concurrency which is simply based on data parallelism. The tasks can be distributed through a queue into available processors and executed in parallel, and in many cases there is either zero or minimal cross-talk between the distributed tasks which simplifies the implementation and exploitation of parallelism. In the following section we will focus on the XTT scenario but the scope that will be covered by TomoPy is not limited to XTT. We will come back to this point in the discussion section.

## 4. Case study: XTT   

**Figure 1:**
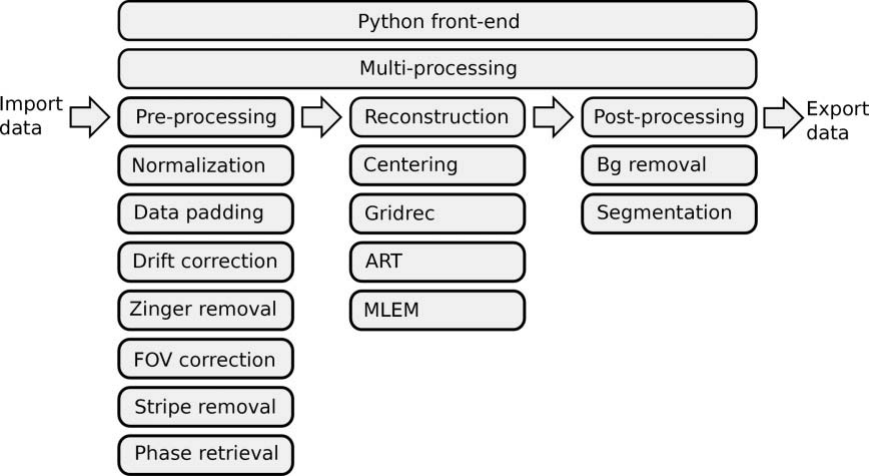
The TomoPy framework. The analysis chain is divided into pre-processing, reconstruction and post-processing modules. Any method or a collection of methods in any language can be hooked to one of these modules without compromising the modularity. A Python front-end is used to interface these modules and interact with the user. A customizable Python-based multiprocessing interface is provided for time-consuming computations.

This section describes the journey of XTT data through multiple transformations, from acquisition to image display. The chain of data processing steps that are highlighted in the following subsections are summarized in Fig. 1 ▶.

### 4.1. Pre-processing modules   

In synchrotron tomography, the imaging sample is placed in the beam path and the X-ray intensity profiles on the detector (also called projections) are acquired by rotating the sample during X-ray exposure. Almost all data acquisition protocols require another set of data taken in the absence of the sample which is usually referred to as the white-field measurements (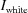) and in the absence of X-ray exposure which is called the dark-field measurements (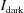). These three sets of measurements together with the rotation angle information are the entry point of the analysis pipeline.

Once the data and the corresponding projection angles are imported, a set of default methods is available under the pre-processing module simply to prepare data for reconstruction. Generally the pipeline of transformations start with data normalization which include dark-field (offset) and white-field (gain) corrections to the raw intensity data (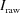), 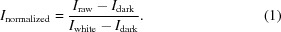
This step is essential not only to compensate different sensitivities and responses in each detector pixel but also to scale the images between 0 and 1 to obtain reliable attenuation information about the sample according to the Beer–Lambert law.

**Figure 2:**
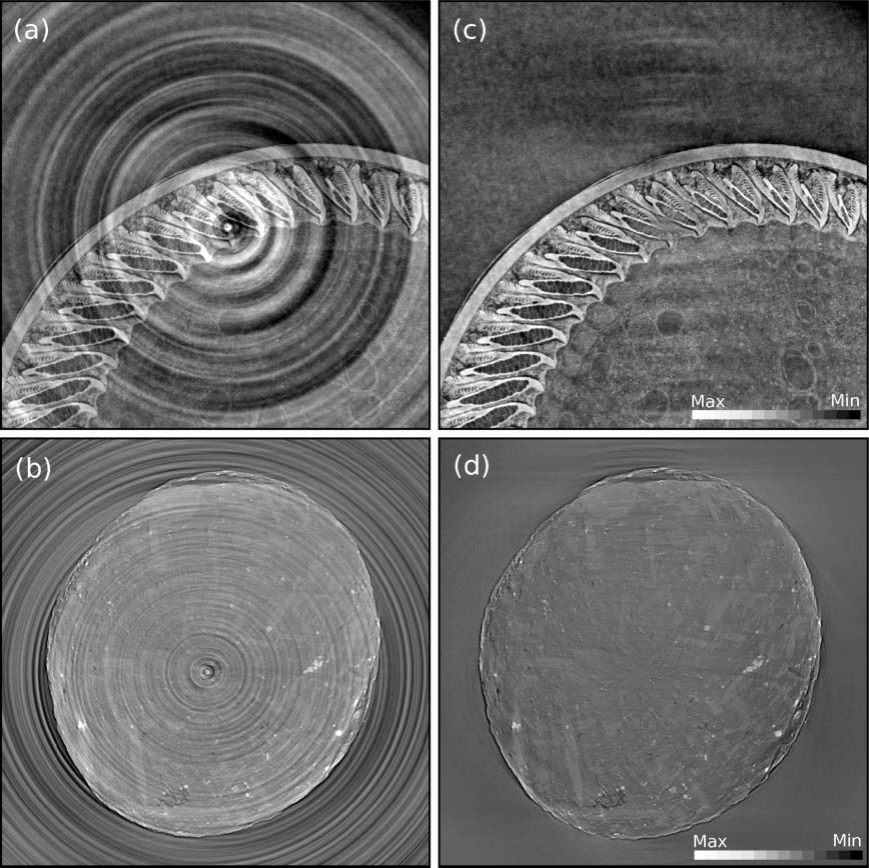
Reconstructed images of a bird feather (*a*–*c*) and a shale rock samples (*b*–*d*) before (*a*–*b*) and after (*c*–*d*) the combined wavelet-Fourier method for stripe removal has been applied on data.

For most datasets, a simple normalization cannot satisfactorily correct for the detection artifacts, which are usually caused by the drift of the inhomogeneous X-ray beam or imperfection of the imaging detector system. Such artifacts appear as stripes in the sinogram domain and as rings in the image domain when the non-ideal normalization persists on some specific pixels in the projection images within an angle range. This is particularly an issue when defects are present on the scintillator screen or on the detector (bad pixel or non-linearity of the pixel response). To correct for these ring artifacts, a combined wavelet-Fourier filtering (Münch *et al.*, 2009 ▶) is implemented in TomoPy. The filtering is essentially based on a set of transformations to condense the artifacts into a tiny region so that removal of them would not cause significant deformations to actual features in the sample and is superior to many other filtering approaches in terms of robustness and conservation of image features (see Fig. 2 ▶).

**Figure 3:**
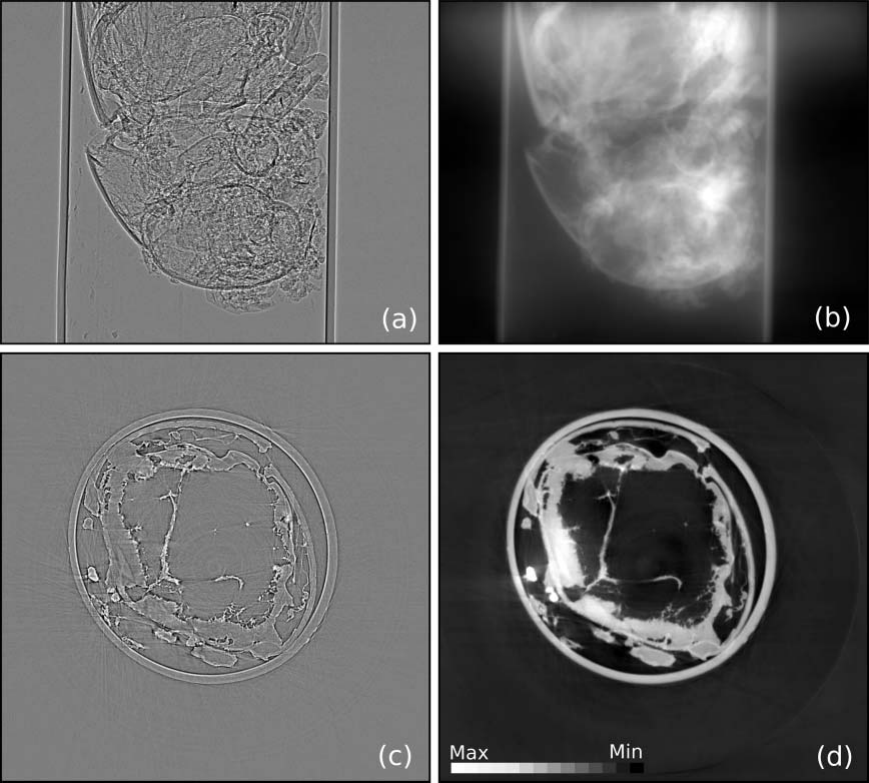
Phase-contrast projection data of an ant placed in a capillary (*a*) lead to a recovered phase image obtained with the single-step phase-retrieval method using a Pagannin filter (*b*). The corresponding reconstructed images of a single slice without and with phase retrieval are shown in (*c*) and (*d*), respectively.

For some samples that are either beam sensitive or have weak absorption contrast (such as biological specimens), an in-line phase-contrast imaging (PCI) mode, which is related to Fresnel diffraction, can be applied (Davis *et al.*, 1999 ▶). For experiments requiring high temporal resolution, a single-distance PCI is of primary importance because it allows much faster scanning speed without the need to alter the detector position multiple times during data acquisition (Burvall *et al.*, 2011 ▶). PCI also may be utilized to separate the phase and the attenuation contrast, and ultimately to reconstruct the distribution of the real and imaginary part of the refractive index separately. TomoPy currently implements Paganin-type (Paganin *et al.*, 2002 ▶) and Bronnikov-type (Bronnikov, 1999 ▶) filters for single-distance phase retrieval. Fig. 3 ▶ shows the phase-contrast data and the corresponding retrieved phase using the Paganin approach.

**Figure 4:**
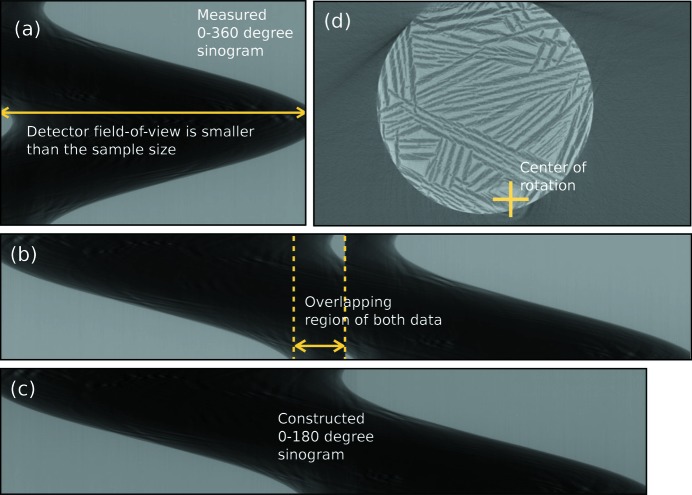
(*a*) Raw 0–360° sinogram of a large sample (porous yttriz-stabilized zirconia) that is not fitting in the detector’s field-of-view. (*b*) Paired 0–180 and 180–360° sinograms. (*c*) Registered sinogram using phase correlation. (*d*) Reconstructed image from the registered sinogram.

Another factor limiting the tomographic reconstruction quality that is addressed by TomoPy is when the sample is larger than the detector’s field of view and the structure of the sample cannot be reconstructed accurately. The artifacts in the reconstruction tend to be stronger when the ratio between the sample size and the detector’s field of view is larger. One way to extend the detector’s field of view is to set the rotation axis at one side of the detector and scan the sample in 360°. The field of view in such an approach is almost double the detector’s native field of view. To reconstruct the sample structure, the measured data are converted to a 0–180° dataset by first pairing 0–180 and 180–360° sinograms and exploiting phase correlation to complete the registration in an automated fashion (see Fig. 4 ▶).

### 4.2. Reconstruction modules   

The next module in the pipeline is the reconstruction module which contains functions that map data from data space into image space. There are numerous methods suitable for this task, each having different strengths and weaknesses depending on the applied data and timing constraints (Mirone *et al.*, 2014 ▶; Rivers, 2012 ▶). As a default method, TomoPy provides Gridrec (Dowd *et al.*, 1999 ▶), which is a direct Fourier-based method that relies on discrete Fourier transforms of data similar to the filtered-backprojection method (Kak & Slaney, 2001 ▶). The main difference is that Gridrec samples a slice in the Fourier domain on a Cartesian grid before transforming back to the spatial domain. The utilization of fast Fourier transforms on a Cartesian grid outperforms other methods in terms of computational speed and is usually desired for quick reconstructions.

**Figure 5:**
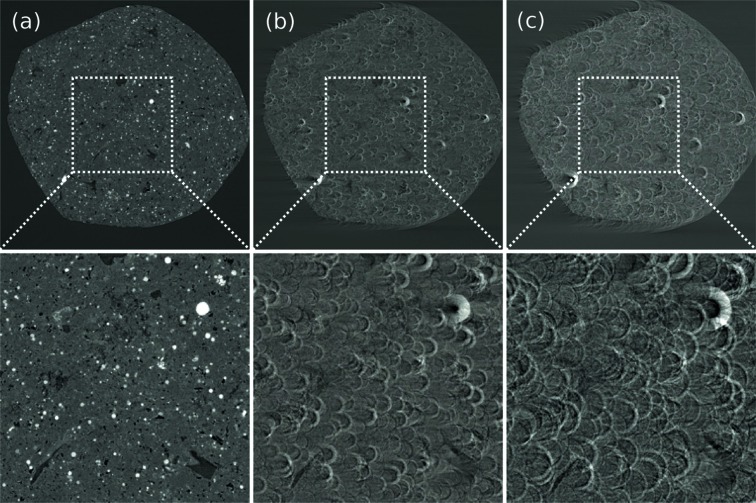
Reconstructed images of a shale rock sample obtained with different centers of rotations: (*a*) correct center, (*b*) 16 pixels off-center horizontally, (*c*) 32 pixels off-center horizontally.

**Figure 6:**
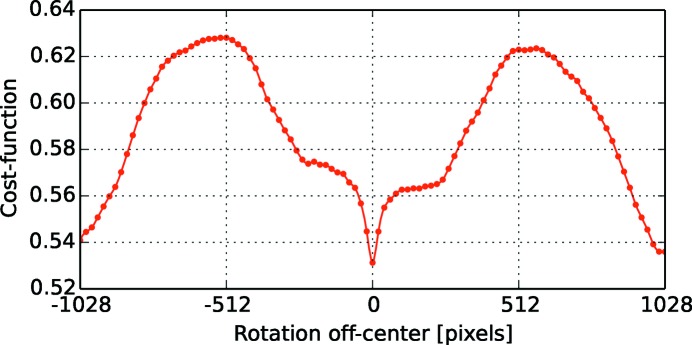
Plot of Shannon entropy as a function of different rotation centers.

The success of the reconstructions usually requires a good estimate of several geometrical parameters such as the location of the rotation center. This problem is unique to synchrotron radiation set-ups, where the X-ray beam and detector are fixed and the sample is rotated. One common way to estimate rotational center positions is to calculate the distance of the sinogram’s center of mass to the mid-point (Azevedo *et al.*, 1990 ▶). Although this method is computationally cheaper than the alternatives, its applicability is only limited to uniformly sampled 360° datasets which makes it less usable in most X-ray absorption tomography applications. Another approach is to exploit the systematic artifacts in reconstructed images due to shifts in the rotation center (Donath *et al.*, 2006 ▶) (see Fig. 5 ▶) as errors to be reduced within an optimization framework. To this end, we utilize Shannon entropy as a measure to evaluate the image quality with the corresponding cost function, 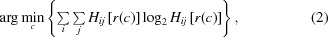
where *c* is the unknown rotation center, 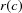 is the reconstructed image which is a function of *c*, and *H* is the histogram of 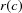. The optimization problem is usually well behaved and can be rapidly solved using any suitable optimization method. As an example, in Fig. 6 ▶ we show the cost function for different rotation centers. A simplex search algorithm converges to the correct rotation center usually in less than 20 iterations for a wide range of initial points which makes it a method of choice if robustness is desired. The central point of the image as an initial guess is generally more than enough for many datasets to find an accurate center in an automated fashion. This reconstruction-based approach to correct for unknown geometrical parameters can also be utilized for example to determine the tilt angle of the rotation axis.

**Figure 7:**
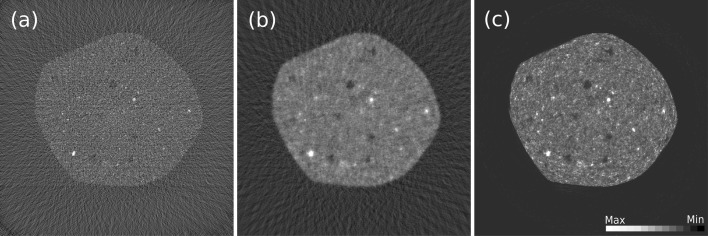
Reconstructed images of a shale rock sample obtained with Gridrec (*a*), ART (tenth iteration) (*b*) and MLEM (50th iteration) (*c*) methods using 46 projections out of an available 1500 projections.

One key component in the TomoPy reconstruction module is the integration and availability of iterative model-based inversion methods. Such methods commonly pose higher computational requirements but generally outperform direct Fourier-based reconstruction methods, especially when the data are under-sampled (few projections available) or suffer from low signal-to-noise-ratio values (fast-scans) which are usually common for XFM and XDT data acquisitions. A comparative demonstration between Gridrec and iterative reconstruction methods is given in Fig. 7 ▶. Iterative model-based methods try to utilize data fidelity based on a system model, and in principle require an accurate forward model which mainly relies on an efficient ray-tracing implementation (*i.e.* computation of the transmission matrix coefficients). To serve this purpose, we have developed a three-dimensional ray-tracing algorithm and computation of the associated transmission matrix coefficients that one can use to construct any iterative reconstruction method. Currently, variants of the algebraic reconstruction technique (ART) (Gordon *et al.*, 1970 ▶) and maximum-likelihood expectation maximization (MLEM) (Dempster *et al.*, 1977 ▶; Lange & Fessler, 1995 ▶) methods for transmission and emission datasets are implemented in TomoPy as alternatives to Gridrec. We also provide models for various imaging components such as the stages, detectors and source characteristics, to be available in case an accurate forward model is required.

### 4.3. Post-processing modules   

For some applications, further processing steps such as segmentation of regions or a quantification analysis may be desired. Several commercial software packages like *Amira* or *Avizo* (FEI Visualization Sciences Group) can provide comprehensive visualization and analysis tools, but for many basic tasks simpler open-source tools are often sufficient. In this framework, we provide integration of a number of post-processing methods from Python’s *Scikit-Image* package (http://scikit-image.org), like segmentation of regions and quantitative analysis of the reconstructions and the sample. The success of an automated segmentation is highly sensitive to the preceding transformations applied on the data. TomoPy implements novel pre-processing methods like the combined wavelet-Fourier filtering model-based inverse models, and improved regularization methods, providing high-quality reconstructed data which allows a relatively easier segmentation step.

## 5. Discussions   

In this paper we introduced methods associated with XTT but the ultimate goal of TomoPy is to become a software framework able to integrate and standardize the available data analysis methods for all synchrotron tomography techniques. Considering the strong similarities among the tomographic techniques, we are designing TomoPy using a modular strategy so that the common methods can be inherited making their functionality available to other techniques (*e.g.* removal of outliers, determination of geometrical parameters, tomographic reconstruction, *etc*.) and specific methods are added as bound to the particular technique.

One common problem of XFM, XDT, Fresnel-zone-plate-aided XTT and other nano-probing techniques is that the imaging resolution is usually finer than the ‘motor resolution’. This causes spatial blurring in the reconstructions and in many cases has to be corrected in manual or semi-automated ways. Our current effort is to develop robust methods to correct for such geometrical aberrations before the reconstruction process begins. One solution would be to incorporate fast phase-correlation methods to roughly align the projections taken from the same instrument initially and apply more sophisticated intensity-based image registration schemes (Maes *et al.*, 1997 ▶) to adjust the geometry at a finer scale. We believe that such entropy-based methods that perform very well with datasets having different contrasts will also pave the way for a comprehensive multi-modal analysis of datasets.

The use of GPU computing for the solution of large-scale tomography problems is becoming increasingly the focus of attention. However, their relatively small built-in memory space and poor data transfer rates limit their use for large datasets. Current implementations in TomoPy are so far for CPU computing but one can easily integrate any GPU-based code using a thin Python wrapper. This modularity allows one to access the full range of capabilities and features of the toolbox (*e.g.* pre-processing functions) in addition to the integrated algorithms using different computing resources.

Many essential processing functions are multi-threaded in TomoPy and the computation speed scales almost linear­ly with the increased number of processing cores. For instance, obtaining reconstructed values from a 1024 × 1024 × 1024 (float32) sized phase-contrast dataset including data import and export takes about less than 4 min on a 24-core workstation (Intel Xeon CPU E5-2620). The most time-consuming processes are: ring removal (84 s), phase retrieval (95 s) and image reconstruction with Gridrec (20 s).

One of the main obstacles of such integration is the lack of a common data format, but recent efforts to adopt HDF5 files with a defined schema (http://aps.anl.gov/DataExchange/) has the potential to significantly ease the integration of data analysis methods (De Carlo *et al.*, 2014 ▶). Currently, TomoPy with the Data Exchange module can import data from other synchrotron facilities including the Advanced Light Source, the Advanced Photon Source, Anka, the Australian Synchrotron, Diamond Light Source, the European Synchrotron Radiation Facility, Elettra, the National Synchrotron Light Source and Petra III.

## 6. Conclusion   

This paper gives a brief glimpse of the methods currently available in the TomoPy framework, but more importantly it emphasizes the importance of unifying the efforts towards the development of data analysis and reconstruction tools targeting synchrotron tomography applications. The success of such initiative, of course, depends on many factors, the most critical of which is our ability to share data and more importantly software tools. We believe the advancements in desktop computer performance, the faster than ever developments of Python packages and the many initiatives bridging applied mathematicians and experimental physicists create the right set of ingredients to establish stronger collaboration and provide ultimately faster deployment of innovative numerical methods.
